# P-65. Results from A Phase 2 Clinical Trial for Treatment of Bone And Joint Infections with Afabicin, A First-in-Class Selective Anti-Staphylococcal Antibiotic

**DOI:** 10.1093/ofid/ofae631.272

**Published:** 2025-01-29

**Authors:** Alireza Shamaei Tousi, Moritz Marquardt, Annick Menetrey, Justyna Nowakowska, Justine Dao, David Cameron, Charlotte Catala-Goldschmidt, Ricardo Chaves

**Affiliations:** Debiopharm International, Lausanne, Vaud, Switzerland; Debiopharm International, Lausanne, Vaud, Switzerland; Debiopharm International, Lausanne, Vaud, Switzerland; Debiopharm International, Lausanne, Vaud, Switzerland; Debiopharm International, Lausanne, Vaud, Switzerland; Debiopharm International, Lausanne, Vaud, Switzerland; Debiopharm International, Lausanne, Vaud, Switzerland; Debiopharm International, Lausanne, Vaud, Switzerland

## Abstract

**Background:**

Staphylococci are the most common causative pathogens in bone and joint infections (BJI). Treatment for BJI is a major clinical challenge, with recurrent and persistent infections occurring in 40% of patients. Afabicin is the most advanced FabI inhibitor in clinical development and has the potential to be the first microbiome-sparing anti-staphylococcal antibiotic. Currently, a Phase 2 trial in BJI (NCT03723551) is enrolling patients to be treated for 3-6 weeks with afabicin or standard of care (SoC). Results of the first cohort in this study (2-3 weeks treatment) are presented here

**Methods:**

In an ongoing multicenter, open-label, Phase 2 study, the safety, tolerability, and efficacy of afabicin was compared to those of SoC, in the treatment of patients with BJI. Patients with osteomyelitis, septic arthritis, or prosthetic joint infections were randomized (5 afabicin: 1 SoC) to receive IV and oral treatment for 14 to 21 days. Afabicin treatment (55 mg IV/ 80 mg PO BID) was compared to a pre-defined SoC treatment.

**Results:**

Twenty patients were included in microbiological intent-to-treat (mITT) population and were treated for up to 21 days with afabicin or SoC (17 afabicin and 3 SoC). The mean treatment duration was 20.1 days for afabicin and 19.7 days for SoC. Overall, osteomyelitis was the most common diagnosis (85%). The most frequent baseline pathogen was *Staphylococcus aureus* (19 patients), and 18 patients had methicillin-sensitive *S. aureus*. All 20 patients were treatment responders at End of Treatment (EoT) visit. The clinical response at 4 weeks post EoT was 13 of 15 patients in the afabicin arm, consistent with patient exposure being above PK/PD targets derived from non-clinical modeling. All 3 patients in the SOC arm achieved clinical response at 4 weeks post EoT. At 12 weeks post EoT, the clinical response rate was 13 of 15 patients in the afabicin arm and 2 of 3 patients in the SoC arm. A similar safety profile was observed in the afabicin arm versus SoC arm.

**Conclusion:**

The clinical response rate and safety profile of 2-3 weeks treatment of staphylococcal BJI with afabicin was comparable to SoC. These data support exploring longer durations of treatment in the study. Additionally, the impact of Afabicin on BJI patient’s gut microbiota will be explored.

**Disclosures:**

**Alireza Shamaei Tousi, PhD**, Debiopharm International: Salary **Moritz Marquardt, n/a**, Debiopharm International: Salary **Annick Menetrey, n/a**, Debiopharm International: Salary **Justyna Nowakowska, n/a**, Debiopharm International: Salary **Justine Dao, n/a**, Debiopharm International: Salary **David Cameron, PhD**, Debiopharm International S.A.: Salary **Charlotte Catala-Goldschmidt, n/a**, Debiopharm International: Salary **Ricardo Chaves, n/a**, Debiopharm International S.A.: Salary

